# Transcriptome Analysis Revealed the Embryo-Induced Gene Expression Patterns in the Endometrium from Meishan and Yorkshire Pigs

**DOI:** 10.3390/ijms160922692

**Published:** 2015-09-18

**Authors:** Jiangnan Huang, Ruize Liu, Lijie Su, Qian Xiao, Mei Yu

**Affiliations:** 1 Key Lab of Agricultural Animal Genetics, Breeding, and Reproduction of Ministry and the Cooperative Innovation Center for Sustainable Pig Production, Huazhong Agricultural University, Wuhan 430070, China; E-Mails: huangjiangnan@webmail.hzau.edu.cn (J.H.); lrz_2003@163.com (R.L.); sulijie@webmail.hzau.edu.cn (L.S.); xiaoqian2013@sjtu.edu.cn (Q.X.); 2 Institute of Animal Husbandry and Veterinary, Jiangxi Academy of Agricultural Sciences, Nanchang 330200, China

**Keywords:** gene expression pattern, pig, endometrium, Meishan and Yorkshire pigs, microarray

## Abstract

The expression patterns in Meishan- and Yorkshire-derived endometrium during early (gestational day 15) and mid-gestation (gestational days 26 and 50) were investigated, respectively. Totally, 689 and 1649 annotated genes were identified to be differentially expressed in Meishan and Yorkshire endometrium during the three gestational stages, respectively. Hierarchical clustering analysis identified that, of the annotated differentially expressed genes (DEGs), 73 DEGs were unique to Meishan endometrium, 536 DEGs were unique to Yorkshire endometrium, and 228 DEGs were common in Meishan and Yorkshire endometriums. Subsequently, DEGs in each of the three types of expression patterns were grouped into four distinct categories according to the similarities in their temporal expression patterns. The expression patterns identified from the microarray analysis were validated by quantitative RT-PCR. The functional enrichment analysis revealed that the common DEGs were enriched in pathways of steroid metabolic process and regulation of retinoic acid receptor signaling. These unique DEGs in Meishan endometrium were involved in cell cycle and adherens junction. The DEGs unique to Yorkshire endometrium were associated with regulation of Rho protein signal transduction, maternal placenta development and cell proliferation. This study revealed the different gene expression patterns or pathways related to the endometrium remodeling in Meishan and Yorkshire pigs, respectively. These unique DEGs in either Meishan or Yorkshire endometriums may contribute to the divergence of the endometrium environment in the two pig breeds.

## 1. Introduction

A major limitation for increasing the litter size in pigs is the prenatal mortality that mainly occurs during the time of attachment (around gestational day (GD) 12–25) and mid-gestation (day 50–70 of gestation) [1,2]. The multiparous Chinese Meishan pigs had a 20%–34% greater prenatal survival than the less prolific Yorkshire pigs [3,4,5,6]. It is generally accepted that a greater number of embryo survival in prolific Meishan pigs is due, in part, to more gradual changes in the uterine milieu and greater uterine capacity [7,8,9,10,11].

The pig is a species with a non-invasive placenta and the uterine luminal epithelium (LE) is intact throughout pregnancy [12,13]. The embryo implantation process is accompanied with transition in uterine luminal epithelial cell polarity from a high to less polar state and secretion of glandular epithelium (GE) [14,15,16]. At the time of implantation, the conceptuses produce estrogen, which acts as a signal for maternal recognition of pregnancy in pigs [17]. Porcine conceptuses secrete increased levels of estrogens on Days 11 and 12 and between Days 15 and 25 of pregnancy [18]. The estrogen, type I interferons (IFNG) and type II interferons (IFND) from the conceptuses together with the progesterone (P4) can temporally and spatially regulate the endometrial gene expression to determine the functional and structural changes in uterine cells [19,20,21]. After GD30, embryo loss are thought to result from the intrauterine crowding in swine caused by the limitations in uterine capacity [22]. Chinese Meishan pigs exhibit physiological strategy by markedly increasing in number of the uterine glands or vascular density and permeability to overcome potential limits in uterine capacity [4,11]. The functional difference between Meishan and Yorkshire uteri is due to the differences in endometrial structural modifications [23,24]. Thus, gaining an understanding of the molecular mechanisms underlying the role of the uterine endometrium remodeling during pregnancy would be an important step towards investigating the molecular basis of the sow prolificacy.

Some studies which were conducted on the investigation of the gene expression profiles in the porcine endometrium during the early gestation phase were published [12,25,26,27,28,29,30,31,32,33,34]. However, investigation of the genes with different expression patterns in endometrium of pigs with prolificacy (Meishan pigs) and less prolificacy (Yorkshire pigs) is an important step towards understanding the mechanism of the uterine function in embryo development and survival. The aim of this study was to identify the differentially expressed genes in endometrium between GD15 and GD26 and between GD26 and GD50 in Meishan and Yorkshire pigs, respectively. We found the common differentially expressed genes (DEGs) in Meishan and Yorkshire gilts related to the signaling networks and pathways which could be essential for endometrial remodeling in response to pregnancy during the early and mid-gestation. Moreover, the identified DEGs unique to Meishan and Yorkshire gilts, respectively, will be useful to investigate the different mechanisms involved in supporting the embryo implantation and growth.

## 2. Results

### 2.1. Detection of the Differentially Expressed Genes

**Figure 1:**
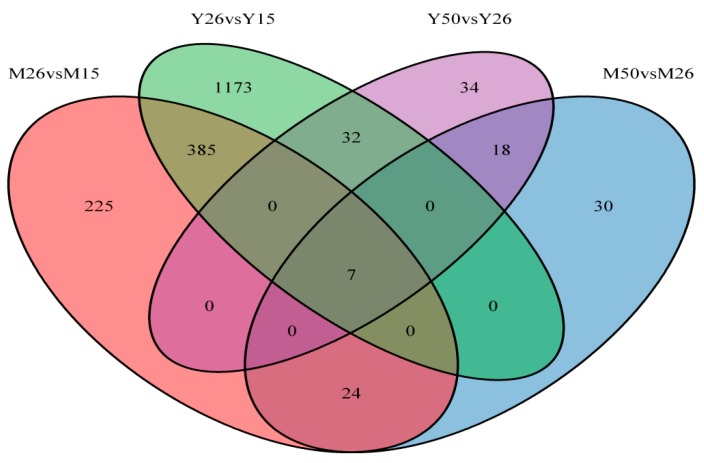
Venn diagram illustration of endometrial genes changed between days 15 and 26 of gestation and days 26 and 50 of gestation in Meishan and Yorkshire pigs. M15, Meishan pigs on day 15 of gestation. M26, Meishan pigs on day 26 of gestation. M50, Meishan pigs on day 50 of gestation. Y15, Yorkshire pigs on day 15 of gestation. Y26, Yorkshire pigs on day 26 of gestation. Y50, Yorkshire pigs on day 50 of gestation. DEGs as defined by FDR < 0.05 and FC ≥ 2.

In pigs, prenatal mortality occurs in several stages of gestation and mainly results from the early embryo mortality and the uterine capacity limits [35]. In order to identify genes expressed in endometrium that may affect the porcine embryo implantation and placentation, the gene expression profiles on GD15, GD26 and GD50 in endometriums of Meishan and Yorkshire gilts were examined, respectively. GD15 and GD26 represent the middle and end of the peri-implantation period (approximately Days 12–25 of pregnancy), respectively. On GD50, the attachment between the pig conceptus trophoblasts and the endometrial epithelial cells is finished and the pig placenta is in a steady-state stage of development [36]. We performed two comparisons (GD26 *vs.* GD15 and GD50 *vs.* GD26) within each breed, respectively. Taking a FC ≥ 2 and the adjusted *p*-value ≤ 0.05 significance level as the criteria, totally, 689 and 1649 annotated genes (DEGs) showed differential expression in endometrium of Meishan and Yorkshire breeds at the three gestational stages, respectively (Table S1 and Table S2). Of the 689 DEGs in Meishan endometrium, 641 genes were found to be differentially expressed between GD15 and GD26 and 79 genes were found to be differentially expressed between GD26 and GD50. Of the 1649 DEGs in Yorkshire endometrium, 1598 genes were found to be differentially expressed between GD15 and GD26 and 91 genes were found to be differentially expressed between GD26 and GD50 (Figure 1). All data related to this study has been deposited on the GEO database (Accession number GSE51787). By hierarchical clustering analysis, we found that 228 DEGs were common in Meishan and Yorkshire gilts, which were likely associated with the embryo implantation and placentation (Table S3), a total of 73 DEGs were unique to Meishan gilts (Table S4) and 536 DEGs were unique to Yorkshire gilts (Table S5).

### 2.2. Identification of Genes Required for the Embryo Implantation and Placentation in Porcine Endometrium

**Figure 2:**
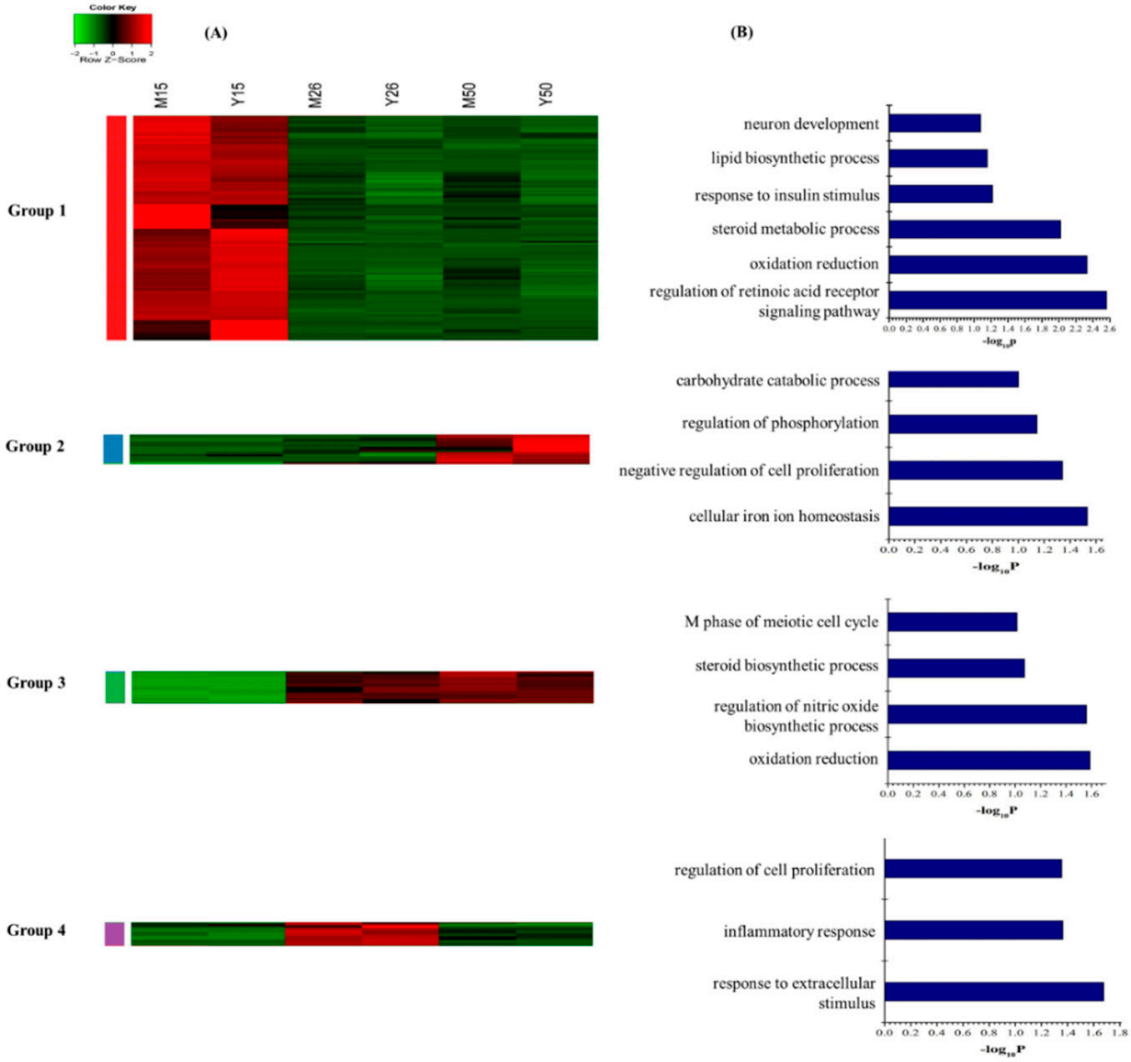
Dynamic progression of the common DEGs in the Meishan and Yorkshire endometrium. (**A**) Unsupervised hierarchical clustering of the 228 annotated common DEGs in the Meishan and Yorkshire endometrium. The common DEGs were clustered into four groups. Red region, genes up-regulated in the endometrium; green region, genes down-regulated in the endometrium. M15, Meishan pigs on day 15 of gestation; M26, Meishan pigs on day 26 of gestation; M50, Meishan pigs on day 50 of gestation; Y15, Yorkshire pigs on day 15 of gestation; Y26, Yorkshire pigs on day 26 of gestation; Y50, Yorkshire pigs on day 50 of gestation; (**B**) Functional categories distribution of the common DEGs in the Meishan and Yorkshire endometrium.

The unsupervised hierarchical clustering analysis revealed that the 228 annotated common DEGs in Meishan and Yorkshire samples were classified into four groups (Figure 2A). (I) Expression level decreased only from GD15 to GD26 and thereafter remained constant (*n* = 167 genes, group 1); (II) Expression level only increased from GD26 to GD50 (*n* = 21 genes, group 2); (III) Expression level increased from GD15 to GD26 and thereafter remained constant (*n* = 23 genes, group 3); and (IV) Increased from GD15 to GD26, then decreased sharply on GD50 (*n* = 17 genes, group 4). The 167 genes in group 1 were enriched for GO terms related to steroid metabolic process, oxidation reduction, response to insulin stimulus, and regulation of retinoic acid receptor signaling pathway. Genes that are involved in negative regulation of cell proliferation and regulation of phosphorylation were found in group 2. The genes in group 3 were enriched for GO terms involved in steroid biosynthesis and genes that regulate the inflammatory response and cell proliferation were in group 4 (Figure 2B, Table S6).

**Figure 3:**
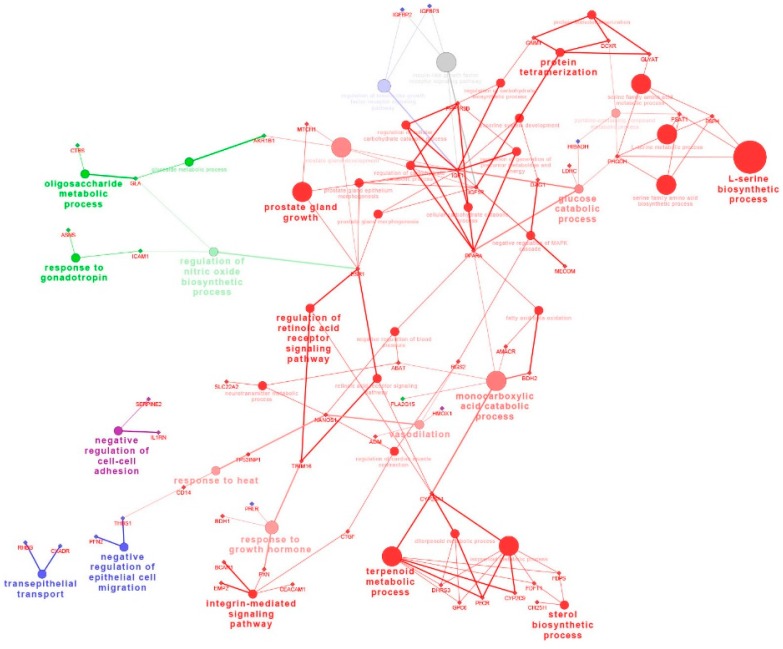
The network of the common DEGs in Meishan and Yorkshire endometrium on gestational day 15, 26 and 50.The genes are represented as diamond. Biological processes are represented as circles. The lines represent the potential connections between the different genes belonging to the different biological process. Red nodes represent genes that have intensive expression on gestational day 15. Blue nodes represent genes represent genes that have intensive expression on gestational day 50. Green nodes represent genes that have intensive expression on gestational day 26 and 50. Purple nodes represent genes represent genes that have intensive expression on gestational day 26.

The KEGG pathways with the most representations for the common DEGs were retinol metabolism (genes in group 1), p53 signaling pathway (genes in group 2), androgen and estrogen metabolism (genes in group 3), or steroid hormone biosynthesis (genes in group 4), respectively (Table S7). To further discuss whether these DEGs in corresponding pathway can be mapped on the Cytoscape network, we analyzed the DEGs extracted in each pathway, respectively. A total of 63 out of the 228 annotated common DEGs were mapped on the Cytoscape network in regulation of the retinoic acid receptor signaling pathway (CYP26A1, TRIM16), the integrin-mediated signaling pathway (BCAR1, CEACAM1, EMP2, PXN, CTGF),the sterol biosynthetic process (CH25H, FDFT1, FDPS) and the negative regulation of epithelial cell migration (THBS1, PFN2), respectively (Figure 3).

### 2.3. Pregnancy Causes Distinct Transcriptome Changes between Early and Mid-Gestation in Meishan Endometrium

**Figure 4:**
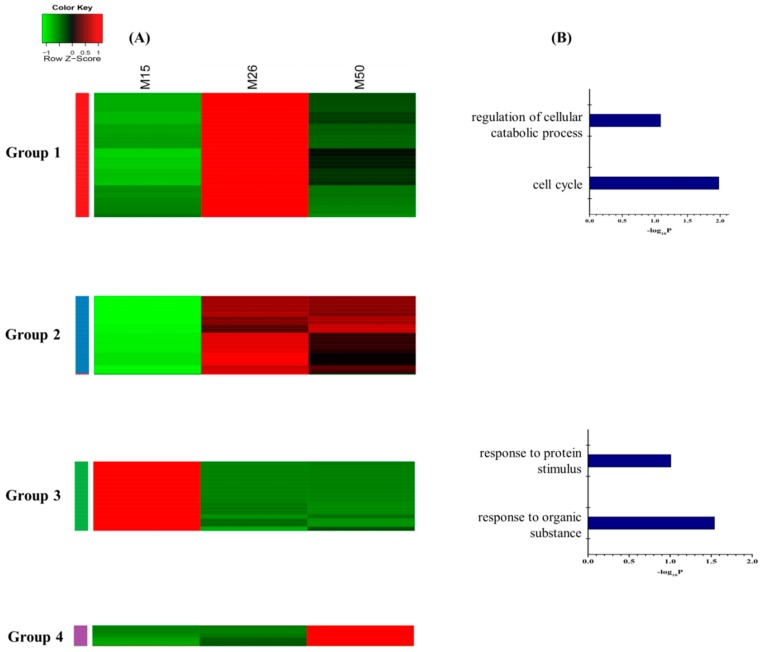
Dynamic of unique DEGs in Meishan endometriums. (**A**) Unsupervised hierarchical clustering of the 73 DEGs unique to Meishan endometrium. DEGs were clustered into four groups. Red region, genes up-regulated in the endometrium, green region, genes down-regulated in the endometrium. M15, Meishan pigs on day 15 of gestation; M26, Meishan pigs on day 26 of gestation; M50, Meishan pigs on day 50 of gestation; (**B**) Functional categories distribution of the unique DEGs in the Meishan endometrium.

**Figure 5:**
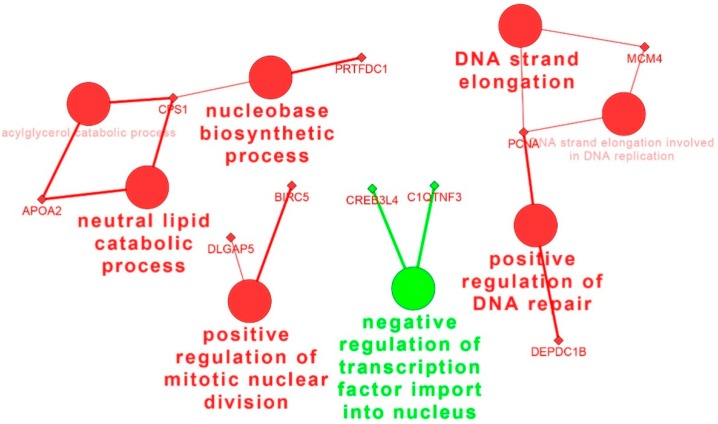
The network of the DEGs unique to Meishan endometrium on gestational days 15, 26 and 50. The genes are represented as diamond. Biological processes are represented as circles. The lines represent the potential connections between different genes belonging to different biological process. Red nodes represent genes up-regulated on gestational day 26. Green nodes represent genes highly expressed on gestational day 15.

Hierarchical clustering based on the 73 unique DEGs in Meishan endometrium displayed four distinct expression patterns (Figure 4A). The group 1 includes the genes with the expression levels increased from GD15 to GD26 and decreased at GD50 (*n* = 31 genes). The group 2 includes the genes with the expression levels increased from GD15 to GD26 and then remained constant (*n* = 19 genes). The group 3 consists of the genes with the expression levels decreasing from GD15 to GD26 and then remaining constant (*n* = 18 genes). The expression pattern of the fourth group was that the gene expression levels only increased from GD26 to GD50 (*n* = 5 genes). The enriched GO terms related to the cell cycle were specifically overrepresented for the genes in the group 1. Furthermore, GO terms related to the response to organic substance were present in the group 3 (Figure 4B, Table S8). No significant annotation terms were identified for DEGs in groups 2 and 4. According to the KEGG database, the most significantly enriched pathways were the cell cycle and adherens junction (Table S9). The network analysis showed that PCNA (proliferating cell nuclear antigen), BIRC5 (baculoviral IAP repeat containing 5), MCM4 (minichromosome maintenance complex component) as the candidate genes might play important roles in regulation of Meishan uterine function during the early and mid-gestation (Figure 5).

### 2.4. Pregnancy Causes Unique Alteration in the Transcriptome of Yorkshire Endometrium

**Figure 6:**
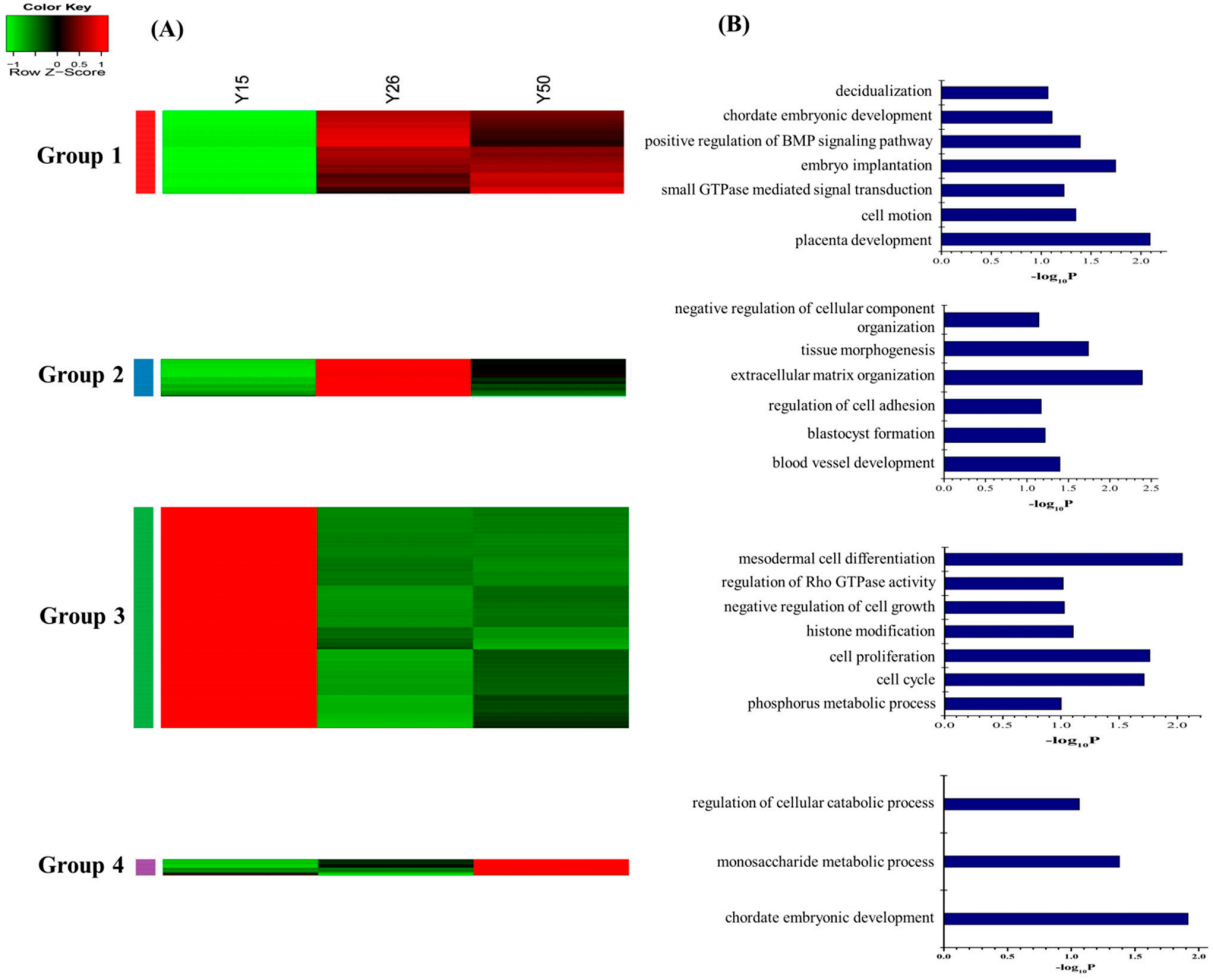
Dynamic progression of the unique DEGs in Yorkshire endometrium. (**A**) Unsupervised hierarchical clustering of 536 differentially expressed genes unique to Yorkshire endometrium. DEGs were clustered into four groups. Red region, genes up-regulated in the endometrium; green region, genes down-regulated in the endometrium. Y15, Yorkshire pigs on day 15 of gestation; Y26, Yorkshire pigs on day 26 of gestation; Y50, Yorkshire pigs on day 50 of gestation; (**B**) Functional categories distribution of unique DEGs in the Yorkshire endometrium.

**Figure 7:**
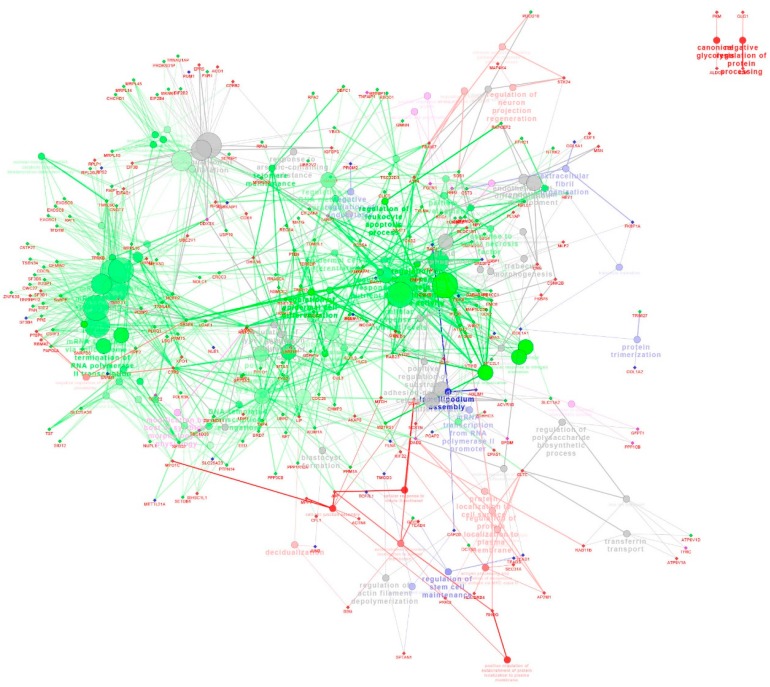
The network of the DEGs unique to Yorkshire endometrium on gestational days 15, 26 and 50. The genes are represented as circles. Biological processes are represented as diamonds. The lines represent the potential regulation relationships between genes or connections between different genes belonging to different biological process. Red nodes represent genes that have intensive expression on gestational day 26 and 50. Blue nodes represent genes that have intensive expression on gestational day 26. Green nodes represent genes that have intensive expression on gestational day 15. Purple nodes represent genes that have intensive expression on gestational day 50.

Consequently, the 536 DEGs, which were unique in Yorkshire gilts, were classified into four groups by hierarchical clustering analysis (Figure 6A). The first group showed an expression level increased from Day 15 to 26 of gestation then remained unchanged at GD50 (*n* = 109 genes). The second group showed an expression level increased from Day 15 to 26 of gestation and then decreased at GD50 (*n* = 56 genes). The third group showed an expression level decreased from Day 15 to 26 of gestation and thereafter remained constant (*n* = 349 genes). The fourth group showed an expression level increased only from Day 26 to 50 of gestation (*n* = 22 genes). GO terms enriched by the DEGs in the group 1 were associated with regulation of Rho protein signal transduction, maternal placenta development and chordate embryonic development. The genes in the group 2 were related to vasculature development, blastocyst formation and regulation of cell adhesion. The genes in the group 3 were involved in mesodermal cell differentiation, cell proliferation and cell cycle. The genes in the group 4 were involved in chordate embryonic development (Figure 6B, Table S10). As shown in Table S11, the significant signaling pathways include tight junction, regulation of actin cytoskeleton, focal adhesion, MAPK signaling pathway and endocytosis (Table S11). Across the Cytoscape network analysis, the genes in the group 1 were enriched in pathways such as salivary gland morphogenesis and apical junction assembly. The genes in the group 3 were enriched in pathways such as the intracellular estrogen receptor signaling pathway, gland morphogenesis and regulation of Ras GTPase activity (Figure 7).

### 2.5. Verification of the Gene Expression Patterns by Quantitative RT-PCR

**Figure 8:**
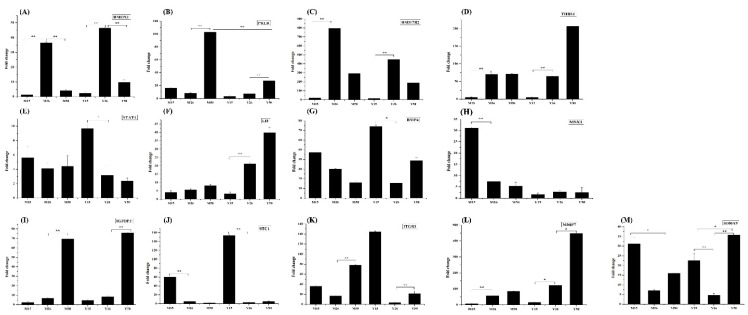
Validation of the expression levels of 13 representative genes by quantitative RT-PCR. The *x*-axis represents the different stages and breeds and the *y*-axis shows the fold changes in expression (** *p* < 0.01, * *p* < 0.05). (**A**) *HMOX1* gene; (B) *PRLR* gene; (**C**) *HSD17B2* gene; (**D**) *THBS1* gene; (**E**) *STAT1* gene; (**F**) *LIF* gene; (**G**) *BMP4* gene; (**H**) *MSX1* gene; (**I**) *IGFBP3* gene; (**J**) *STC1* gene; (**K**) *ITGB3* gene; (**L**) *MMP7* gene; (**M**) *S100A9* gene.

Ten DEGs were selected to validate the results of the microarray analysis by quantitative RT-PCR. Of the selected DEGs, four DEGs were identified as common in Meishan and Yorkshire endometrium (*THBS1*, *HMOX1*, *PRLR* and *HSD17B2*), one DEG was unique to Meishan endometrium (*MSX1*), three DEGs were identified as unique to Yorkshire endometrium (*STAT1*, *LIF* and *BMP4*) and two DEGs (*MMP7* and *S100A9*) were expressed differentially in at least one comparison in both Meishan and Yorkshire endometriums. The expression patterns of all the 10 selected DEGs obtained by quantitative RT-PCR were consistent with the results from the microarray analysis (Figure 8). In addition, three DEGs (*STC1*, *ITGB3* and *IGFBP3*) that showed common expression patterns in Meishan and Yorkshire endometriums but did not reach the statistically significant level in the microarray analysis were also selected for validation due to their roles in remodeling of endometrium. The results confirmed the common expression patterns of the three DEGs in Meishan and Yorkshire endometrium (Figure 8).

## 3. Discussion

In this study, the large numbers of DEGs identified in both Meishan and Yorkshire endometriums between GD15 and GD26 (Table S1 and Table S2) revealed that major temporal changes occur in the endometrium transcriptome between the conceptus attachment stage (GD15) and the post-implantation stage (GD26). Additionally, we found that the number of DEGs identified as unique to Yorkshire endometrium is much greater compared to the number of DEGs unique to Meishan endometrium, which could suggest that the uterine environment in Meishan breed may be relatively stable.

The endometrium is a complex steroid responsive tissue. The progesterone and estrogen may constitute a “servomechanism” that regulates the endometrial remodeling (remodeling of the endometrial epithelium and stromal cell) and the uterine receptivity during the early pregnancy in pigs [37,38]. In this study, we identified the genes involved in regulation of retinoic acid receptor signaling pathway (*ESR1*, *CYP26A1*, *TRIM16*) and retinol metabolism-related enzyme genes (*DHRS3*, *CYP2C9*, *CYP26A1*) that were highly expressed in both Meishan and Yorkshire endometriums at GD15. It was found that CYP26A1 might block the adverse effect of the retinoic acid in order to promote the successful embryo implantation [39]. TRIM16, also named estrogen-responsive B box protein and acts as a positive regulator of the CYP26A1 expression, showed a similar expression pattern with *CYP26A1* gene in both Meishan and Yorkshire endometriums. The expression of *ESR1* in the porcine endometrium may indicate direct action of estrogen on proliferation and differentiation of endometrial cells [40]. The estrogen and retinoic acid signaling pathway provides regulation of “Yin and Yang” in cell proliferation and survival [38,41]. These results reminded us that except the progesterone and estrogen, the interaction between the retinoic acid and estrogen signaling may be also important for the embryo–maternal communications and endometrium remodeling during the early pregnancy.

Interestingly, we found increased expression of genes in steroid hormone biosynthesis pathway (*CYP3A7*, *HSD17B2* and *SULT1E1*) in GD26 endometrium (Figure 2A, Group 4) and genes encoding androgen and estrogen metabolic enzymes (*HSD17B12* and *HSD11B1*) in both GD26 and GD50 endometrium (Figure 2A, Group 3). These genes play important roles in synthesis and metabolism of progesterone and estrogens. The imbalance between the actions of estrogen and progesterone are associated with abnormal uterine bleeding and proliferative disorders [42]. Sulfotransferases could convert estrogens into non-active estrogen sulphates [33] and the expression of P450 aromatase in the pregnant mouse uterus plays an essential role in converting testosterone into E_2_ [43].Thus, the increased expression of *SULT1E1* and *CYP3A7* genes in the pregnant endometrium suggested that the regulator to balance the ratio of progesterone to estrogen might be the fine tuning estrogen levels in the uterus. Above all, these results indicated that during the early and mid-gestation, the gene involved in adjusting uterine steroid and retinol concentration may be essential for embryo implantation and growth.

Previous evidence suggests that the endometrial morphology and uterine function differs during the gestational stages in pigs [35]. It has been proposed that the acquisition of the uterine receptivity results from the loss of epithelial cell polarity [44,45]. The factors that mainly attribute to the uterine epithelial cell function appear to play a central role in mediating endometrial receptivity. The transcript level of the *Msh homeobox 1* gene, *Msx1*, was decreased uniquely in Meishan endometrium from GD15 to GD26 and then remained at a low expression level in GD50. As the negative regulator of the cell differentiation, *Msx1* was expressed in mouse uterine luminal epithelial cell at the time of the pre-implantation phase and declined at time of implantation and post-implantation phases [46,47,48]. Further investigation indicated that *Msx1* gene functions in regulation of the transition in uterine luminal epithelial cells from a high to a less polar state and thus leading to a transition of uterus from the pre-receptive to the receptive phase [49]. Thus, compared to Yorkshire pigs, the higher expression of *Msx1* gene at implantation stage in Meishan endometrium implied a reduced uterine epithelial polarity to make the uterus conducive to blastocyst attachment to the luminal epithelium (LE) at the onset of implantation.

The genes involved in cell cycle, such as *CCNB2*, *NCAPG*, *DLGAP5*, *BIRC5*, *CDC20*, *TACC1* and *PCNA*, were up-regulated in the post-implantation stage (GD26) and then decreased at GD50 in Meishan endometrium. The cell cycle regulators are critical for controlling the stromal cell proliferation and differentiation during the implantation stage [50]. *PCNA* (proliferating cell nuclear antigen) is a cell cycle promoting gene which was observed primarily in uterine luminal epithelium and glandular epithelium at pre-implantation stage and its expression level was increased gradually in stromal cells and myometrium with progressing gestation [51,52,53]. This increased expression of cell cycle-related genes at the time of Meishan embryo implantation suggested that the cell proliferation and differentiation in Meishan endometrium were associated with the embryo attachment. Interestingly, gene ontology analysis using group 3 DEGs in Yorkshire endometrium (genes that have intensive expression at GD15) revealed that cell proliferation and cell cycle were among the most significantly enriched biological processes (Figure 6B, Table S10). Compared to group 3 in Yorkshire endometrium (Figure 6) with the group 1 in Meishan endometrium (Figure 4), we found that the cell cycle and cell proliferation regulators showed the opposite expression patterns between Meishan and Yorkshire endometrium during the embryo implantation stage (GD15). Taking into account the above, the genes that showed higher expression levels in endomtrium of Meishan pigs on GD15 and GD26 may influence the uterine receptivity by regulating the uterine cell phenotype and contribute to promoting the endometrium remodeling in Meishan pigs during the early pregnancy.

Compared to the Meishan breed, the decreased embryo survival in Yorkshire breed was suggested to be due to a promotive effect of the uterus on embryonic development and growth [6,54,55]. We found the genes that were associated with regulation of the maternal placenta development (*LIF*, *BSG*) and the chordate embryonic development (*FKBP8*, *EPAS1*, *TEAD4*, *CFL1*, *SPINT1*, *CDH1*) have intensive expression in Yorkshire endometrium at GD26 and GD50.The epithelial marker E-cadherin was found to be localized in luminal and glandular epithelial cells during the peri-implantation period of pregnancy [56]. Its encoding gene, *CDH1*, was up-regulated from GD15 to GD26 and then remained constant at GD50 (Figure 6). At the same time, we also detected the down-regulated expression of some genes which are involved in suppressing the expression of *CDH1* gene, such as the transcriptional repressor of E-cadherin gene (*SIP1*, also known as *ZEB2*), and *PTEN* and *GSK3B* genes [57,58]. A previous report found that the down-regulation of *SIP1* resulted in reduced invasion and migration of cells, along with the up-regulation of *CDH1* [59]. The up-regulation of *CDH1* also resulted in the increasing of the epithelial cell polarity and glandular formation by inducing mesenchymal-to-epithelial transition (MET) [60,61,62]. It has been indicated that the polarized uterine epithelial cells could secrete a large number of cytokines and growth factors to promote conceptus elongation and development [63,64]. These unique DEGs to Yorkshire endometrium may dynamically regulate uterine epithelial cell polarity, promote secretion of the endometrial gland, thereby stimulating the blastocyst growth and development.

## 4. Experimental Section

### 4.1. Animals and Tissue Collection

All the experimental and surgical procedures complied with the Guide for Care and Use of Laboratory Animals and were approved by the Biological Studies Animal Care and Use Committee of Hubei Province, China. The Meishan and Yorkshire gilts of similar age and weight were observed twice a day for estrous behavior using intact boars. On the day of first standing oestrus (Day 0) and 24 h later, Meishan and Yorkshire gilts from Jingpin farm of Huazhong Agricultural University were mated to boars of their respective breed. The first day of mating was considered to be Day 0 of gestation. Pregnancy was confirmed by the presence of normal conceptus in the uterine flushing (Day 15) or at hysterectomy (Days 26 and 50). Uteri were obtained from animals slaughtered on Days 15, 26 and 50 of gestation (*n* = 2 gilts//breed/gestational day). Tissue collection were according to the descriptions by Su *et.al* (2014) [65]. The endometrial tissues were placed in liquid nitrogen immediately and stored at −80 °C until extraction of RNA.

### 4.2. Porcine Affymetrix GeneChip Hybridization and Data Analysis

Total RNA was extracted from the frozen tissues using Trizol reagent (Invitrogen, Carlsbad, CA, USA) according to the manufacturer’s instructions. In order to prevent contamination of genomic DNA, RNase-free DNase kit (Qiagen, Valencia, CA, USA) was used according to the manufacturer’s manual. Total RNA concentrations and quality were determined by using a NanoDrop ND-2000 Spectrophotometer (Thermo Scientific, Wilmington, DE, USA). Then, the amount of each RNA sample used for subsequent microarray analysis was made to be equal in a unit volume. As described by Su *et al*. (2014) [65], for each breed, RNA sample from one endometrial site of one gilt was pooled in equal volume with that from the corresponding site of the other gilt on each gestational day. Thus, three pools of RNA samples from three different conceptus-attachment sites of two gilts on each gestational day for each breed were used for the microarray hybridization. The RNAs were sent to a commercial service for hybridization to the Porcine AffymetrixGenechip^®^ (Affymetrix, Santa Clara, CA, USA).

The raw data intensity files were read into R [66] and preprocessed using functions of the Affy and GCRMA packages of the BioConductor project [67]. Probe sets whose intensity was above 100 in at least six arrays were retained and then subjected to identification of the significantly differentially expressed genes by the linear model and the empirical Bayes methods (Limma R package). The genes expressed by the endometrium with significant changes at the three different gestational stages were identified in each breed and the comparisons of GD26 *vs.* GD15 and GD50 *vs.* GD26 were made within each breed. The raw *p*-values were adjusted for multiple testing using the Bonferroni and Hochberg false discovery rate methods. Lists of DEGs were selected on the basis of an adjusted *p*-value of ≤0.05 and fold change (FC) ≥2. DEGs were considered to be unique to Meishan (Yorkshire) pigs if they obtained an adjusted *p*-value <0.05 and FC ≥ 2 in Meishan (Yorkshire) pigs dataset but *p*-value >0.05 and FC < 1.5 in Yorkshire (Meishan) pigs dataset. Probe annotation was firstly done using the file supplied by Tsai *et al*. [68]. The target sequences of the probe sets were confirmed by BLAST in porcine genome as well as other genomes including human, mouse and bovine. In total, 23,985 (99% of all) probe sets were assigned RefSeq annotation. All microarray data have been deposited into the NCBI Gene Expression Omnibus and are accessible through GEO Series accession number GSE51787 [69].

### 4.3. Clustering Analysis

The expression values for the differentially expressed genes obtained by averaging the intensity values of analogous probe sets on the microarray were subjected to unsupervised hierarchical clustering analysis by correlation similarity matrix and complete linkage algorithms using R language.

### 4.4. Gene Ontology (GO) and KEGG Pathway Analysis

Gene Ontology (GO) enrichment analysis was performed by tools available at DAVID Bioinformatics Resources to detect the overrepresented functional gene categories [70,71]. The list of differentially expressed genes was uploaded into KEGG (Kyoto Encyclopedia of Genes and Genomes) [72] to identify the statistically significantly enriched biological pathways in which the differentially expressed genes participated. The network was built by Cytoscape [73].

### 4.5. Quantitative RT-PCR for DEGs Validation

Quantitative RT-PCR was used to verify the differential expression of 13 genes that were detected by the Affymetrix GeneChip. Total RNA from three endometrial samples from each gilts (*n* = 4 gilts/gestational day) was reversely transcribed using SuperScript II Reverse Transcriptase and Oligo(dT) (Invitrogen, Carlsbad, CA, USA) according to the manufacturer’s instructions. The quantitative RT-PCR was performed on 36 endometrial samples in total (attachment sites/gilts/breeds/gestational days) for each gene to be validated. The primers were listed in Table S12. Each real-time RT-PCR reaction (in 25 μL) contained 2× SYBR Green Real-time PCR Master Mix (Toyobo, Japan), 0.4 μM primers, and 0.5 μL of template cDNA. The cycling conditions consisted of an initial, single cycle of 5 min at 95 °C, followed by 40 cycles of 30 s at 95 °C, 30 s at 60 °C, 15 s at 72 °C, and melting curves were obtained by increasing the temperature from 58 to 95 °C at 0.5 °C/s for 10 s. The PCR amplifications were performed in triplicate for each sample. The LightCycler 480 Software 1.5 (Roche, Risch, canton of Zug, Switzerland) was used to obtain *C*_t_ values. Relative quantification analyses were done in EXCEL using the comparative *C*_t_ method. The gene expression levels were quantified relative to the expression of *ACTB* by employing an optimized comparative *C*_t_ (ΔΔ*C*_t_) value method. The differences in gene expression levels between groups were compared using the Student’s *t*-test. A *p*-value ≤0.05 was considered significant.

## 5. Conclusions

Collectively, the study revealed the common differentially expressed genes in endometrium on days 15, 26 and 50 of gestation in Meishan and Yorkshire pigs which were likely essential for the embryo implantation and placentation in pigs. Findings regarding the genes which were unique to either Meishan or Yorkshire pigs could suggest that different genes or pathways might be involved in endometrial remodeling in two pig breeds during early and mid-gestation.

## Supplementary Materials

Supplementary materials can be found at http://www.mdpi.com/1422-0067/16/09/22692/s1.
