# Supplementary material for: Transcriptome Analysis Revealed the Embryo-Induced Gene Expression Patterns in the Endometrium from Meishan and Yorkshire Pigs
Source: Int J Mol Sci. 2015 Sep 18;16(9):22692–710. doi: 10.3390/ijms160922692 (PMC4613331; doi:10.3390/ijms160922692)
Supplement: Supplementary file 1 [file ijms-16-22692-s001.zip › ijms-95264-Supplementary Information/Table S12.pdf]

Supplementary Information

Table S12. Primers used for quantitative RT-PCR.

Gene	Primer	Primer Sequences (5′–3′)	Primer Location	Tm (°C)	Size (bp)
THBS1	THBS1-F	AGCAGCCGTTTCTATGTT	Exon 19–20	60	220
	THBS1-R	AATCTTTCCAGCCTATGTG	Exon 20		
HMOX1	HMOX1-F	CGCTCCCGAATGAACACT	Exon 3	60	137
	HMOX1-R	TGGTCCTTAGTGTCCTGGGT	Exon 4		
PRLR	PRLR-F	CTTGGTGGTGGCTGCGTC	Exon 10	60	169
	PRLR-R	GGGAGGTCCAGGGATTGT	Exon 10		
HSD17B2	HSD17B2-F	GCATAGTATTTGCTGGAGTTCT	Exon 1	60	244
	HSD17B2-R	CATCGTTTGTATTGGGTCAT	Exon 2		
STC1	STC1-F	TGAGGCGGAGCAGAATGA	Exon 1	60	279
	STC1-R	CGAATGGCGAGGAAGACC	Exon 1		
ITGB3	ITGB3-F	CGGCAGGTGGAGGATTAC	Exon 4	60	248
	ITGB3-R	ATGGGCAAACAGGTGGTCT	Exon5		
IGFBP3	IGFBP3-F	CAGAGCACGGACACCCAGAA	Exon 2	60	225
	IGFBP3-R	CTTATCCACGCACCAGCAGA	Exon 4		
MMP7	MMP7-F	ACACTGTTGCTACACTTCAC	Exon 2	60	140
	MMP7-R	TGCCTGAGGAGAGTCAAGAT	Exon 6		
S100A9	S100A9-F	CAAATGTCGCAGATGGAATG	Exon 2	60	178
	S100A9-R	GGATGTGGTTTATGGCTTTC	Exon 3		
STAT1	STAT1-F	CTGCCAATGATGTTTCGT	Exon 4	60	123
	STAT1-R	TGCTTTTCCTAATGTTATGCT	Exon 4		
MSX1	MSX1-F	TGTGGTTTCTCCTCGGTT	3′-UTR	60	267
	MSX1-R	ATTTGTCTGGGCTTTTCC	3′-UTR		
BMP4	BMP4-F	TAGGAGCCATTCTGTAGTGC	Exon 2	60	228
	BMP4-R	CCCCGTCTCAGGTATCAA	Exon 4		
LIF	LIF-F	GAGCCATTTCCCAACAAC	Exon 2	60	120
	LIF-R	GTAGGCGATGATGCGGTA	Exon 3		
β-actin *	Actb-F	ACGTGGACATCAGGAAGGAC		60	210
	Actb-R	ACATCTGCTGGAAGGTGGAC			

* β-actin serves as the control gene for the endomtrium genes.
